# Loss of the PTH/PTHrP receptor along the osteoblast lineage limits the anabolic response to exercise

**DOI:** 10.1371/journal.pone.0211076

**Published:** 2019-01-25

**Authors:** Joseph D. Gardinier, Conor Daly-Seiler, Niloufar Rostami, Siddharth Kundal, Chunbin Zhang

**Affiliations:** 1 Bone and Joint Center, Henry Ford Hospital, Detroit, MI, United States of America; 2 Biomedical Physics Department, Wayne State University, Detroit, MI, United States of America; University of California Davis, UNITED STATES

## Abstract

Exercise and physical activity are critical to maintain bone mass and strength throughout life. Both exercise and physical activity subject bone to a unique combination of stimuli in the forms of dynamic loading and a systemic increase in parathyroid hormone (PTH). Although dynamic loading is considered to be the primary osteogenic stimuli, the influence of increasing PTH levels remains unclear. We hypothesize that activation of the PTH/PTH-related peptide type 1 receptor (PPR) along the osteoblast lineage facilitates bone formation and improved mechanical properties in response to exercise. To test this hypothesis, conditional PPR-knockout mice (PPR^cKO^) were generated in which PPR expression was deleted along the osteoblast lineage under the osterix promoter. At 8-weeks of age, both PPR^fl/fl^ and PPR^cKO^ mice were subjected to treadmill running or sedentary conditions for 5-weeks. Under sedentary conditions, PPRcKO mice displayed significantly less bone mass as well as smaller structural-level strength (yield-load and ultimate load), while tissue level properties were largely unaffected. However, PPRcKO mice exposed to exercise displayed significantly less structural-level and tissue-level mechanical properties when compared to exercised PPR^fl/fl^ mice. Overall, these data demonstrate that PPR expression along the osteoblast lineage is essential for exercise to improve the mechanical properties of cortical bone. Furthermore, the influence of PPR activation on material properties is unique to exercise and not during normal growth and development.

## Introduction

The incidence and economic burden of osteoporotic fractures continues to grow each year.[1] Maintaining bone mass and strength are critical to preventing osteoporosis and reducing fracture risk throughout life. Exercise and physical activity throughout life are key preventative measures recommended by the National Osteoporosis Foundation to reduce fracture risk.[2] Understanding the underlying mechanisms by which exercise regulates bone metabolism is essential to developing therapeutic strategies that reduce risk of osteoporosis and fracture.

Both exercise and physical activity subject bone to a unique combination of stimuli in the forms of dynamic loading and a systemic increase in parathyroid hormone (PTH).[3] The increased levels of systemic PTH during exercise represents the 84–amino acid sequence of intact PTH secreted by the parathyroid gland, and occurs at the onset of exercise, such as running, swimming, or weight-lifting.[3] Both clinical and animal studies have found the release of PTH transient in nature by returning to basal levels within 1 hour of a single exercise session.[4–7] In addition to PTH released by the parathyroid gland, exercise also elicits the local expression of the PTH-related peptide (PTHrP).[8] In-vitro studies have gone on to demonstrate that the release of PTHrP is regulated by dynamic loading, and that the release of PTHrP is a more delayed response compared to the release of PTH by the parathyroid gland.[9] Despite similarities in structure and binding affinity to the PTH/PTHrP type 1 receptor (PPR), the variations in amino acid sequence allow PTH and PTHrP to activate different down-stream mechanisms.[10–12] To understand the contribution of PTH and PTHrP signaling on the osteogenic response to exercise we have previously treated mice with PTH(7–34) to block down-stream PPR activation. Although PTH(7–34) was able to inhibit PPR activation of select anabolic pathways and limit bone adaptation in response to exercise, PTH(7–34) has recently been shown to activate alternative mechanisms that influence bone formation through β-arrestin [13–15] As a result, the extent to which PPR activation across specific cell-types impacts bone adaptation during exercise still remains unclear.

Independent of loading, systemic changes in PTH levels have a significant effect on bone metabolism through activation of the PPR. More specifically, PPR activation in bone stimulates both bone resorption and bone formation that is dependent on which cell-types are activated and the temporal pattern of activation.[16] For example, deleting PPR expression under the osteocalcin promoter expressed by both osteocytes and osteoblasts decreases trabecular bone formation, while deleting PPR expression predominately in osteocytes under the dentin-matrix protein 1 promoter produces an opposite effect.[17–19] Furthermore, PPR activation in the kidney can also shift bone remodeling by stimulating 1,25(OH)2D3 production, the most potent metabolite of vitamin D.[20] Vitamin D is key stimulator of osteoclastogenesis and bone resorption by activating osteoblasts expression of the receptor activator of nuclear factor kappa-B ligand.[21,22] Conversely, increased vitamin D levels also enhance osteoblast differentiation and deposition of new tissue, producing a shift in bone remodeling that favors bone formation.[22] As a result, the release of PTH during exercise has the potential to indirectly effect bone metabolism. However, the relative contribution of each mechanism to bone adaptation in response to exercise is still unclear.

Altogether, previous studies demonstrate that the systemic increase in PTH during exercise has the potential to influence bone metabolism, and that direct or indirect effect of PTH may be mediated in a tissue and cell specific manner. We hypothesize that direct PPR activation along the osteoblast lineage facilitates bone formation and improved mechanical properties in response to exercise. To test this hypothesis, PPR expression was deleted in mice along the osteoblast lineage using the osterix (Osx) promoter to determine its role in facilitating bone adaptation in response to treadmill exercise.

## Methods

### 2.1. In-vivo protocols

Animal procedures were conducted under Institutional Animal Care Use Committee (IACUC) approval at the Henry Ford Hospital. To target PPR expression along the osteoblast lineage, mice expressing a doxycycline inducible cre-recombinase under the osterix promoter (Osx-Cre^TetOff^, kindly provided by Dr. Yuji Mishina) were crossed with mice in which the E1 exon of the PPR gene is flanked by Lox-P sites (PPR^fl/fl^, kindly provided by Dr. Henry Kronenberg). The resulting heterozygous mice were back-crossed with PPR^fl/fl^ mice to generate the homozygous Osx-Cre^TetOff^:PPR^fl/fl^ mice (PPR^cKO^). Both Osx-Cre^TetOff^ and PPR^fl/fl^ strains had been back-crossed on a C57Bl6J background for more than 10 generations. All mice were fed chow supplemented with doxycycline (TD.110720, Envigo) to suppress cre-recombinase prior to the experiment at a daily dose of ~2mg/kg body weight. The Osx-Cre genotype was determined based on PCR of tail DNA using the forward Cre primer (5’-CGCGGTCTGGCAGTAAAAACTATC-3’) and reverse Cre primer (5’-CCCACCGTCAGTACGTGAGATATC-3’) as described in literature.[23] PPR-floxed alleles were identified by the sequence spanning the 3’ lox-P site using the forward (5’-TGGACGCAGACGATGTCTTTACCA-3’) and reverse primers (5’-ACATGGCCATGCCTGGGTCTGAGA-3’) established in literature.[24] The PPR^fl/fl^ mice served as controls and were fed the same chow as all other mice.

At 7-weeks of age, the doxycycline was removed from the diet of both PPR^fl/fl^ and PPR^cKO^ mice. At 8-weeks of age, both PPR^fl/fl^ and PPR^cKO^ mice were divided into sedentary and exercise groups. Each group consisted of 10 male mice. Female mice were not used due to lack of numbers. Exercise groups were subjected to treadmill running on a 5 degree incline at 12 m/min for 30 minutes each day and 5 days each week. Both sedentary and exercise groups received 2 fluorochrome injections of calcein green (15 mg/kg) on day 2 and day 31 to quantify the degree of mineralization and mineral apposition rate during the course of the experiment. After 5 weeks of exercise and sedentary treatment, mice were euthanized to isolate the tibiae, which is known to exhibit greater adaptation compared to the femur [25]. The left tibia was used for histomorphometry, and the right tibia for micro-CT analysis and mechanical testing.

### 2.2. Micro-computed tomography (μCT) analysis

Prior to mechanical testing, the cortical bone architecture of the tibia was measured using a custom-built μCT system previously described [26]. Each tibia was first embedded in 1% agarose and placed in a plastic tube to avoid dehydrating the tissue. Ex-vivo scans were then taken with the following settings: 16 μm voxel size, 60 kVp, 0.5 mm aluminum filter, 83 μA, and 720 views over 360 degrees, with each view averaging 4 frames. Images were reconstructed using a greyscale threshold optimized across all the samples and then oriented to match their position during mechanical testing. Cortical bone architecture was evaluated at a standard site and the site of fracture. The standard site was defined midway between the loading points. At both sites, cortical bone thickness, cross-sectional area, distance from the most lateral surface to the neutral axis, and moment of inertia about the anterior-posterior axis (MOI_A/P_) were determined.

### 2.3. Histomorphometry

Fluorochrome labels were used to quantify the amount of tissue formed during the experiment. Each tibia was first embedded in methyl methacrylate (Koldmount Cold Mount kit, Mager Scientific, MI) following graded ethanol dehydration. A single section, 250 microns thick, was cut from each sample at the mid-diaphysis using a low-speed sectioning saw (South Bay Technology, Model 650, CA) with a diamond wafering blade (Mager Scientific, MI). Each section was then polished on wet silicon carbide abrasive disks to a final thickness of 100μm. Fluorochrome labels were then identified under a confocal fluorescence microscope (FLUOVIEW FV1000, Olympus) and post-processed using Fiji software. The mineralizing surfaces (MS) was measured as a percentage of the endosteum and periosteum surfaces. The mineral apposition rate (MAR) for each sample was measured by the average distance between double labels. The bone formation rate (BFR) was calculated based on MS and MAR according to standardized histomorphometric analysis.[27] The area of new tissue along the endosteum was measured between the initial calcein label and endosteal surface as described in our previous work.[28] For each sample, the area of new tissue at each surface was normalized to total bone area.

### 2.4. Monotonic fracture test

The mechanical properties were measured for each tibia within 3–4 days of being scanned for μCT analysis. Prior to testing, samples were stored at 4°C. Mechanical testing was performed under four-point bending using the EnduraTech ELF 3200 Series (Bose, MA) as described in our previous work.[8] The tibia was positioned in the loading device with the most distal portion of the tibia and fibula junction directly over the left-most support point and that the medial surface was tested under tension. Each tibia was loaded at a rate of 0.025 mm/s until failure. The load and displacement were recorded and then used to calculate the stress (σ) and strain (ε) relationship based on beam-bending theory using the following equations:

$$\sigma =Fac/2I_{AP}$$

ε=6cd/a(3L‑4a)

where ‘F’ is the recorded force, ‘a’ is the distance between loading supports (3 mm), ‘c’ is the distance from the medial surface to the neutral axis, I_AP_ is the moment of inertia along the anterior-posterior axis, ‘d’ is the measured displacement, and ‘L’ is the span between the outer supports (9 mm). A 0.2% offset of the stress-strain relationship was used to calculate the yield point as described in literature.[29]

### 2.5. Statistical analysis

All outcome measures are reported as the group mean +/- the standard error of mean. For each measurement variable, a two-way analysis of variance (ANOVA) was performed to determine main and interaction effects of genotype (PPR^fl/fl^ vs. PPR^cKO^) and physical activity (sedentary vs. exercise) with repeated measures and Tukey post-hoc testing between groups. Throughout the study, a p-value less than 0.05 was considered significant.

## Results

### 3.1. Loss of PPR expression reduces growth

**Fig 1:**
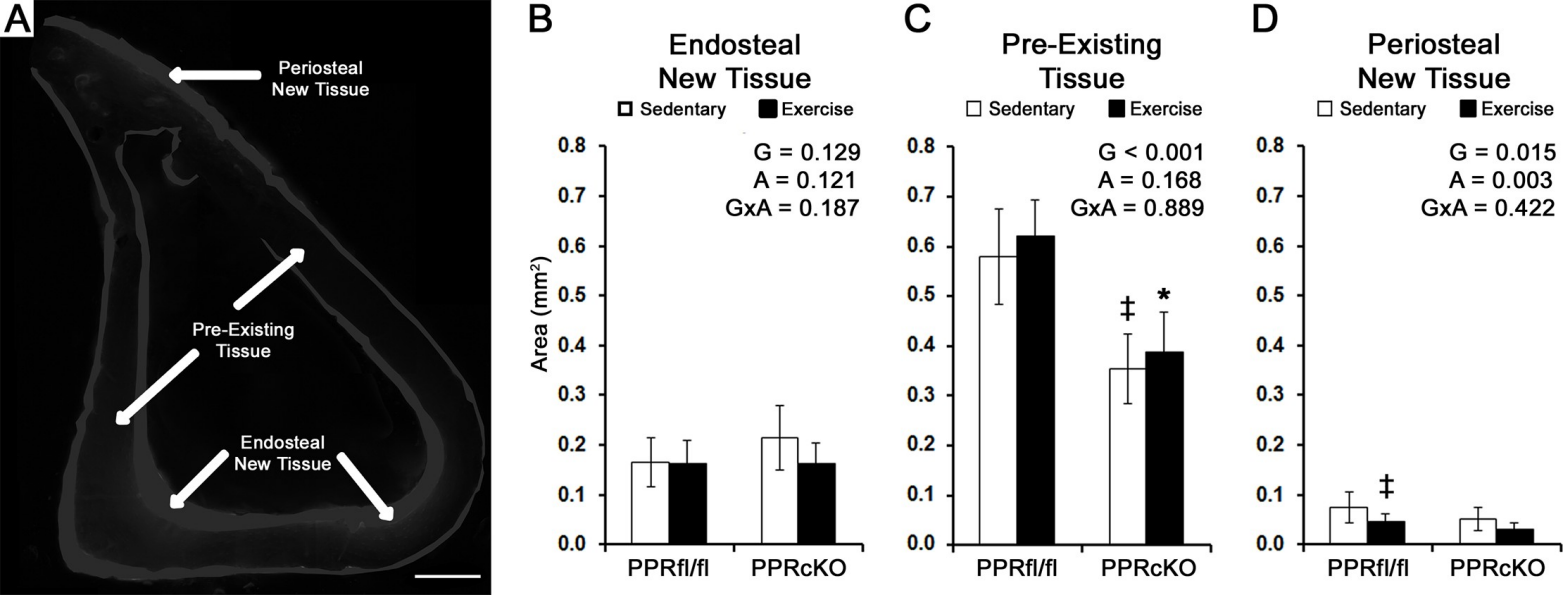
Bone formation at periosteal surface is affected by genotype and exercise during growth. **A**) Representative image of the new tissue identified at the endosteal and periosteal surfaces, along with the pre-existing tissue in-between (bar = 500 μm). The area of tissue between florescent labels was used to quantify **B**) new endosteal tissue, **C**) pre-existing tissue, and **D**) new periosteal tissue. Main effects are noted for genotype (G), physical activity (A), and their interaction (GxA). Individual differences with a p<0.05 were found when compared to sedentary-PPR^fl/fl^ (‡), and exercised-PPR^fl/fl^ (*). Mean ± stdev (n = 10). D).

At 7 weeks of age, prior to removing doxycycline from the diet to induce the knockout, PPR^cKO^ mice were significantly smaller in body weight than PPR^fl/fl^ controls (16.7 ± 2.1 g vs. 23.1 ± 1.0 g, p<0.05). At 13-weeks of age sedentary PPR^cKO^ mice still exhibited a significantly smaller body weight compared to sedentary PPR^fl/fl^ mice (Table 1). Within each genotype, sedentary mice were significantly greater in body weight compared to exercised mice. Genotype had a main effect on tibia length as well as cortical area and MOI_A/P_, each of which was significantly smaller in PPR^cKO^ mice compared to PPR^fl/fl^ control mice (Table 1). Double label histomorophometry demonstrated that both genotype and activity had no significant effect on the MS, MAR, and BFR at both endosteal and periosteal surfaces of the tibia (Table 1). To get a better estimate of how much tissue was produced during the course of the experiment the area of tissue between the fluorescent labels was quantified (Fig 1). At the periosteal surface, the area of new mineralized tissue between the double labels revealed main effects from both genotype and activity (Fig 1D). In particular, PPR^fl/fl^ mice subjected to exercise displayed significantly less new periosteal tissue when compared to sedentary PPR^fl/fl^ mice (0.074 ± 0.06 mm^2^ vs. 0.046 ± 0.013 mm^2^; p<0.05).

**Table 1:** Mouse body weights along with the structural geometry, histomorphometry, and mechanical properties of the tibia in response to physical activity and genotype. Mean ± stdev (n = 10).

	PPR^fl/fl^		PPR^cKO^		Two-Way ANOVA Results		
	Sedentary mean (std)	Exercise mean (std)	Sedentary mean (std)	Exercise mean (std)	Genotype (*p*-value)	Activity (*p*-value)	Interaction (*p*-value)
Body Weight (g)	26.2 (2.4)	24.8 (1.5) ^‡^	20.0 (2.7) ^‡^	17.3 (1.9) *^,^^#^	**<0.001**	**0.019**	0.692
**Geometric Properties**							
Tibia Length (mm)	18.29 (0.54)	18.11 (0.24)	17.55 (0.81) ^‡^	16.92 (0.60) *	**0.002**	0.182	0.371
Cortical Area (mm^2^)	0.75 (0.1)	0.71 (0.07)	0.60 (0.10) ^‡^	0.58 (0.07) *	**<0.001**	0.363	0.808
MOI_A/P_ (mm^4^)	0.079 (0.020)	0.079 (0.011)	0.064 (0.017) ^‡^	0.059 (0.012) *	**0.005**	0.683	0.692
Distance to Neutral Axis (mm)	0.625 (0.039)	0.617 (0.035)	0.595 (0.033)	0.596 (0.038)	0.089	0.884	0.890
**Histomorphometry**							
Endosteal MS/BS (μm/μm)	0.696 (0.182)	0.837 (0.119)	0.823 (0.220)	0.774 (0.222)	0.628	0.499	0.160
Endosteal MAR (μm/week)	19.4 (8.7)	16.6 (4.6)	18.1 (9.4)	11.4 (7.2)	0.232	0.083	0.480
Endosteal BFR (μm/μm/week)	14.5 (8.0)	13.8 (4.4)	16.5 (10.4)	9.7 (6.1)	0.675	0.176	0.261
Periosteal MS/BS (μm/μm)	0.371 (0.096)	0.360 (0.100)	0.336 (0.080)	0.372 (0.114)	0.742	0.732	0.508
Periosteal MAR (μm/week)	4.7 (4.4)	5.8 (4.8)	6.8 (6.0)	4.9 (4.8)	0.754	0.793	0.403
Periosteal BFR (μm/μm/week)	1.96 (1.8)	2.1 (2.0)	2.2 (1.9)	1.7 (1.7)	0.921	0.780	0.650

^‡^ p<0.05 compared to sedentary-PPR^fl/fl^

* p<0.05 compared to exercised-PPR^fl/fl^

^#^ p<0.05 compared to sedentary-PPR^cKO^

### 3.2 PPR expression regulates the structural-level properties of bone and adaptation in response to exercise

**Fig 2:**
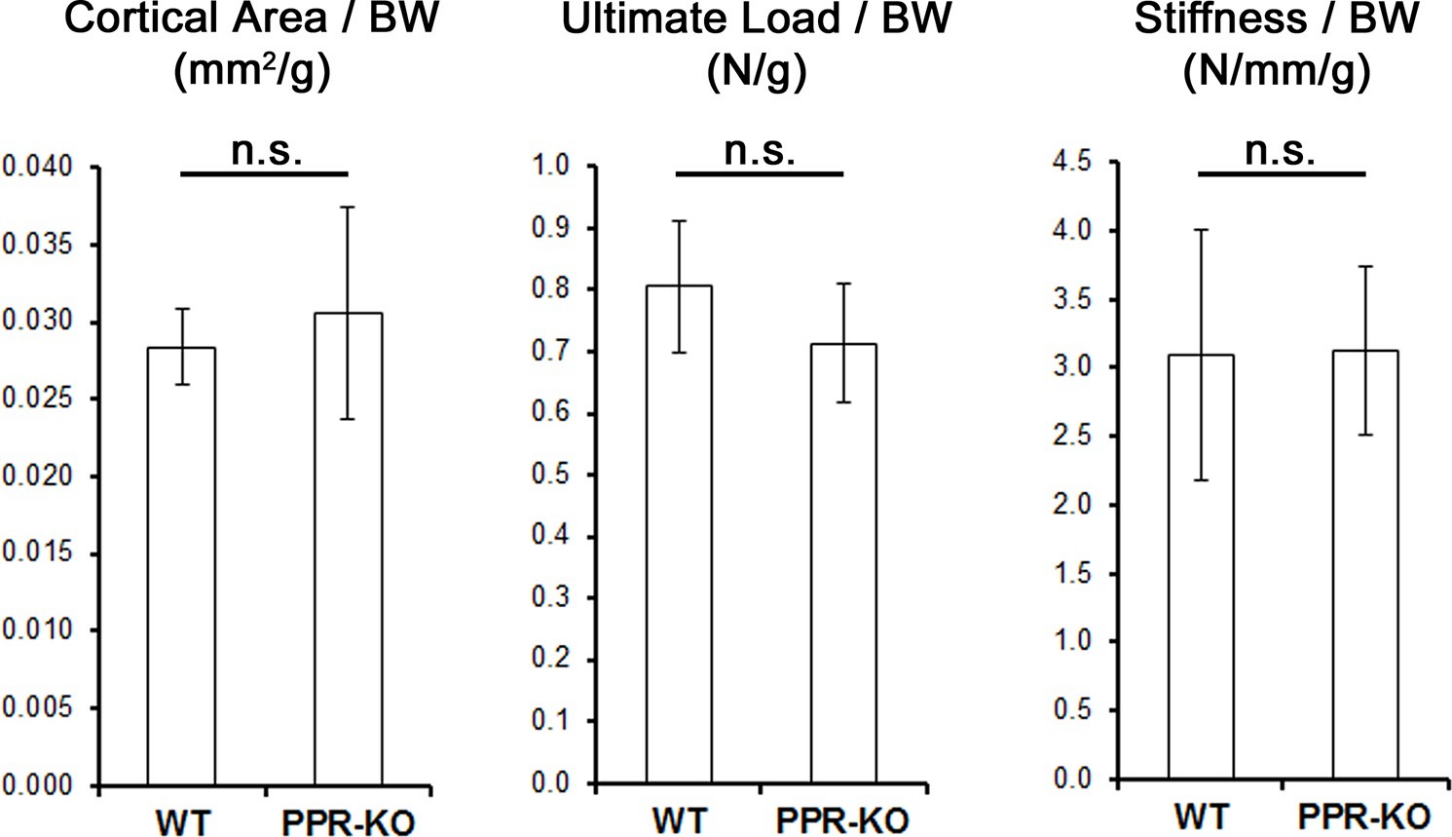
In sedentary mice the cortical area and structural-level properties of the tibia are scaled to body weight between genotypes. Cortical cross-sectional area, ultimate load, and stiffness of sedentary mice were normalized to body weight. Student t-tests found differences between groups not significant (n.s.), suggesting that structural properties are scaled to body weight in the absence of PPR expression. Mean ± stdev (n = 10).

Given the differences in cortical size and geometry, the mechanical properties of the tibia were then measured. Genotype had a significant main effect on the structural-level properties of stiffness, ultimate-load, ultimate-displacement, and pre-yield work, while exercise had a significant main effect on stiffness alone (Table 2). In particular, PPR^fl/fl^ mice displayed significantly greater yield load, ultimate load, and pre-yield work compared to PPR^cKO^ mice across both exercise and sedentary groups. In PPR^fl/fl^ mice alone, exercise produced a significant increase in stiffness compared to sedentary PPR^fl/fl^ mice. Conversely, exercised PPR^cKO^ mice did not display a significant change in stiffness compared to sedentary PPR^cKO^ mice, but was significantly smaller than exercised PPR^fl/fl^ mice. Given the significant differences in growth between genotypes, we normalized the cortical area, ultimate load, and stiffness to body weight and found no significant differences between PPR^fl/fl^ and PPR^cKO^ mice (Fig 2). These findings indicate that in the absence of PPR expression, the structural properties are scaled to body weight.

**Table 2:** Structural-level properties of the tibia are influenced by PPR expression along the osteoblast lineage. Mean +/- stdev (n = 10).

	PPR^fl/fl^		PPR^cKO^		Two-Way ANOVA Results		
	Sedentary mean (std)	Exercise mean (std)	Sedentary mean (std)	Exercise mean (std)	Genotype (*p*-value)	Activity (*p*-value)	Interaction (*p*-value)
Yield Load (N)	19.1 (4.5)	21.1 (2.6)	12.3 (2.1) ^‡‡^	11.4 (2.3) **	**<0.001**	0.600	0.174
Yield Displacement (μm)	297.2 (84.4)	250.1 (50.5)	231.5 (45.1)	217.5 (40.2)	**0.016**	0.126	0.402
Ultimate Load (N)	21.2 (3.7)	22.5 (2.3)	14.1 (1.8) ^‡‡^	13.6 (1.8) **	**<0.001**	0.649	0.322
Ultimate Displacement (μm)	372.8 (98.7)	293.1 (59.4)	381.2 (96.5)	390.5 (61.9) **	**0.056**	0.215	0.112
Stiffness (N/mm)	82.47 (28.6)	109.5 (20.1) ^‡^	61.2 (9.1)	60.3 (9.9) *	**<0.001**	**0.045**	**0.030**
Pre-Yield Work (mJ)	2.96 (1.08)	2.57 (0.59)	1.62 (0.52) ^‡‡^	1.43 (0.48) **	**<0.001**	0.352	0.863

^‡^ p<0.05 compared to sedentary-PPR^fl/fl^

^‡‡^ p< 0.01 compared to sedentary-PPR^fl/fl^

* p< 0.05 compared to exercise-PPR^fl/fl^

** p< 0.01 compared to exercise-PPR^fl/fl^

### 3.3 PPR expression regulates adaptation to tissue-level mechanical properties during exercise

Mechanical testing under four-point bending allowed tissue-level properties to be estimated at the fracture site. Genotype had a main effect on modulus, ultimate-stress, ultimate-strain, and pre-yield toughness, while exercise had a main effect on modulus alone (Table 3). In PPR^fl/fl^ mice, exercise produced a significant increase in modulus compared to sedentary controls. This increase in modulus following exercise did not occur in PPR^cKO^ mice. The exercised PPR^cKO^ mice also displayed significantly smaller modulus, ultimate-stress, and pre-yield toughness when compared to exercised PPR^fl/fl^ mice. In addition, exercised PPR^cKO^ mice displayed significantly greater ultimate-strain compared to exercise PPR^fl/fl^ controls.

**Table 3:** The absence of PPR expression along the osteoblast lineage reduces the impact of exercise on the tissue-level properties of the tibia. Mean ± stdev (n = 10).

	PPR^fl/fl^		PPR^cKO^		Two-Way ANOVA Results		
	Sedentary mean (std)	Exercise mean (std)	Sedentary mean (std)	Exercise mean (std)	Genotype (*p*-value)	Activity (*p*-value)	Interaction (*p*-value)
Yield Stress (MPa)	187.4 (41.7)	234.7 (42.5)	155.3 (54.4)	161.8 (41.6) **	**0.002**	0.088	0.193
Yield Strain (με)	25,540 (6,606)	21,545 (5,238)	20,667 (3,837)	18,629 (3,185)	**0.023**	0.074	0.554
Ultimate Stress (MPa)	209.7 (39.2)	249.9 (41.4)	176.5 (56.2)	192.4 (43.5) *	**0.005**	0.077	0.437
Ultimate Strain (με)	31,867 (6,339)	25,260 (6,227)	33,784 (6,507)	33,747 (7,202) *	**0.022**	0.136	0.140
Modulus (GPa)	9.1 (2.2)	14.0 (2.4) ^‡^	9.0 (4.3)	10.1 (2.9) *	**0.055**	**0.008**	0.076
Pre-Yield Toughness (MPa)	2.50 (0.96)	2.38 (1.16)	1.77 (0.63) ^‡^	1.72 (0.58) *	**0.004**	0.888	0.754

^‡^ p< 0.05 compared to sedentary-PPR^fl/fl^

* p<0.05 compared to exercise-PPR^fl/fl^

** p<0.01 compared to exercise-PPR^fl/fl^

## Discussion

The present findings demonstrate that PTH signaling during exercise has a unique role in bone adaptation that is mediated through PPR activation along the osteoblast lineage. By deleting PPR expression in osteoblasts and osteocytes, we were able to prevent exercise from modifying the material properties of bone based on measured changes in tissue-level mechanical properties. Furthermore, changes in tissue-level mechanical properties through PPR expression was unique to exercise and not growth or development under sedentary conditions. The activation of PPR during exercise is likely in response to either PTH released by the parathyroid gland or PTHrP released locally. In particular, exercise significantly increases osteocytes’ production of PTHrP, which in turn would activate PPR in an autocrine/paracrine fashion.[8] However, Chow et al demonstrated in rats that removing the parathyroid glands abrogates both trabecular and cortical bone formation in response to exogenous loading, suggesting that systemic levels of PTH plays a larger role than the local expression of PTHrP in regulating the sensitivity of bone to dynamic loading.[30] In either case, our findings argue that direct activation of the PPR in bone is responsible for modifying the material properties of bone during exercise. In our previous work, inhibition of PPR activation during exercise by way of treating with PTH(7–34) inhibited trabecular bone growth as well as structural-level mechanical properties, but failed to prevent gains in tissue-level mechanical properties. The inability to inhibit changes to tissue-level properties similar to this study is most likely due to the limited capacity of PTH(7–34) to inhibit PPR activation along with its potential to activate anabolic pathways through β-arrestin.[15] Thus, by deleting PPR expression genetically, the present study demonstrates for the first time that PTH signaling during exercise also plays a critical role in regulating tissue-level mechanical properties.

The increase in modulus following exercise is considered a function of changes in both the extracellular matrix and mineral composition of bone. Similar findings have been reported in response to exercise and attributed to an increase in pyridinoline cross-linking within the extracellular matrix of bone, as well as increase the carbonate-to-phosphate ratio of the existing cortical bone.[13,28,31,32] In addition, decreased mineralization surrounding individual lacuna are also evident following treadmill exercise.[13] Although the exact contribution each adaptation has on the material properties is not clearly defined, we have previously shown changes in mineral composition to be a function of PTH signaling during exercise.[13] Furthermore, the impact of PTH signaling on mineral composition is unique to exercise, given that PTH treatment alone produces differing effects.[33] This may explain why the effect of PPR expression on tissue modulus is only observed during exercise and not normal development. However, the modulus is also likely influenced by changes in the cross-linking profile during exercise. Since PPR activation increases expression of collagenous and non-collagenous proteins,[34] further investigation is warranted to examine the influence of PPR expression on potential changes in matrix composition during exercise.

In agreement with previous studies, exercise had no significant effect on cortical bone size and geometry (Table 1).[8,14,31,32] The lack of changes in cortical size is consistent with the lack of changes in MS, MAR, and BFR at the periosteal and endosteal surfaces. However, the periosteal surface displayed a significant decrease in the area of new mineralized tissue at the periosteum that was not evident based on the calculated BFR (Fig 1D). Given that BFR is only an estimate of how much tissue is formed based on the two scaler values of MS and MAR, the area of new tissue represents a more accurate measure of the tissue volume formed during the course of the experiment. In contrast, we have previously reported the same exercise regimen to increase periosteal BFR and MAR in 8-week old mice despite no significant changes in the periosteal perimeter, cortical area, cortical thickness, or moment of inertia.[8] The inconsistency of BFR between studies is likely due to differences in the basal levels of bone turnover. In our previous studies, the MS, MAR, and BFR was less than half what was found in the present study, which would have allowed the effect of exercise to be more readily observed. The high turnover in our present study has the potential to mask any effects of exercise.[8] Other studies in which bone turnover is extremely high have also shown treadmill exercise to limit bone growth based on reduced cortical area.[28] Together, our findings suggest that during early growth and development when bone turnover is extremely high, exercise may play a larger role in regulating the material properties of the tissue present or that developed at either surface instead of how much tissue is being formed.

Independent of exercise, the loss of PPR expression along the osteoblasts lineage caused a significant decrease in cortical bone growth and tibia length. Similar findings have been reported when only deleting PPR expression in mature osteoblasts and osteocytes that express osteocalcin.[18] Conversely, deleting PPR expression in late osteoblasts/osteocytes has an opposite effect by increasing trabecular and cortical bone formation as mice age.[17,19] As expected the delay in growth corresponded with a significant decline in structural-level properties (Table 2). However, tissue-level mechanical properties were unaffected by the loss of PPR expression (Table 3), suggesting PPR expression does influence the material properties of cortical bone during growth and development. Instead, our findings demonstrate that the effect of PPR activation on material properties is unique to exercise alone.

A key limitation to this study was the disparity between PPR^fl/fl^ and PPR^cKO^ mice at the onset of the experiment. The PPR^fl/fl^ controls consisted of PPR^fl/fl^ mice that were littermates to the PPR^cKO^ mice. However, the Oxs-Cre expressed by PPR^cKO^ mice produced an unexpected phenotype that was not accounted for by the PPR^fl/fl^ mice. The Osx-Cre model used in this study was first developed by Dr. Rodda and Dr. McMahon [35]. Since, only a few studies have reported Osx-Cre expression to produce minor craniofacial defects in the skull as well as delayed cortical growth at the periosteum prior to 6-weeks of age.[36–38] Although the Tet-Off system used to control Osx-Cre expression can abrogate some of these phenotypes, measurable levels of Osx-Cre expression can be observed even when mice are being treated with doxycycline.[36] “Leakage” within the Tet-Off system is common [39–41], and might have contributed to the delayed cortical bone growth in PPR^cKO^ mice by causing PPR expression to be deleted prematurely. A similar delay in bone growth and body weight were reported in a non-inducible model that used the osteocalcin promoter to delete PPR expression in osteoblasts and osteocytes.[18] As a result, the difference in growth between PPR^cKO^ and PPR^fl/fl^ mice is likely a function of either Osx-Cre expression or premature deletion of PPR expression. The impact of these differences on the response to exercise was considered minimal because structural-level mechanical properties scaled to body weight as well as cortical area (Fig 2). Furthermore, the material properties of bone were the same between genotypes, suggesting that the overall mechanical behavior of bone under loading is the also the same between genotypes.

In summary, PPR expression along the osteoblast lineage is essential for exercise to improve the material properties of cortical bone. Furthermore, the influence of PPR activation on material properties is unique to exercise and does not occur during normal growth and development. Although the source of activation was not identified, targeting the osteoblast lineage demonstrated that either PTH release by the parathyroid gland or local PTHrP expression during exercise is directly contributing to bone adaptation. Overall, PPR activation is a critical component for bone adaptation to occur in response to exercise and physical activity.
